# Reply to Pantelis, A.G. Comment on “Salazar et al. Weight Regain after Metabolic Surgery: Beyond the Surgical Failure. *J. Clin. Med.* 2024, *13*, 1143”

**DOI:** 10.3390/jcm13144157

**Published:** 2024-07-16

**Authors:** Juan Salazar, Valmore Bermúdez

**Affiliations:** 1 Endocrine and Metabolic Diseases Research Center, School of Medicine, University of Zulia, Maracaibo 4004, Venezuela; 2 Facultad de Ciencias de la Salud, Universidad Simón Bolívar, Barranquilla 080001, Colombia; valmore.bermudez@unisimon.edu.co

We want to express our sincere gratitude for Dr. Pantelis’ time and interest in reviewing our article and providing valuable feedback. Dr. Pantelis’ insightful comments [1] have undoubtedly helped us improve the quality and accuracy of our work.

Regarding the terms and definitions of “weight regain” and “surgical failure”:

We wholeheartedly agree with your assessment regarding the need to redefine and refocus these concepts in light of the growing recognition of obesity as a chronic disease. As outlined in the ASMBS statement [2], standardizing the nomenclature, similar to that used in oncology, would be crucial to establishing universal and specific definitions in post-surgical care. As you rightly point out, this would allow for more precise epidemiological estimates and facilitate comparisons of outcomes across different studies.

Concerning the complication rates:

It is true that with the advancement of surgical techniques over the years, there has been a significant decline in both short- and long-term complication rates, particularly in reoperations. This fact is corroborated by short- and medium-term analyses of patients undergoing One Anastomosis Gastric Bypass, especially as a primary procedure [3]. 

However, it is noteworthy that the complication rates presented in our review are largely consistent with those reported in recent meta-analyses, such as those by Chungyoon Kim [4] and Balamurugan [5], regarding early complications. In this sense, the Clavien–Dindo classification allows the standardization of definitions of complications.

On the role of bariatric surgery in the “weight set-point”:

While bariatric surgery is considered a factor that can potentially alter the “weight set-point,” it is important to emphasize that, to date, no definitive clinical models substantiate this theory. In this regard, post-bariatric weight recurrence could represent an ideal clinical setting to evaluate the role of the potential mechanisms involved in the regulation of body weight and the gut–brain axis.

Preoperative predictors of weight recurrence:

Although our review primarily focuses on the post-operative mechanisms of weight recurrence, it is important to acknowledge the existence of various preoperative predictors, including genetic predisposition, preoperative weight loss, and insulin sensitivity, among others [6]. In this context, the preoperative metabolic phenotype emerges as a potential predictor to consider in the clinical setting.

Once again, we appreciate your valuable comments and hope this response has addressed your concerns. We remain at your disposal for any further inquiries.
